# Estimated cost-effectiveness of early screening strategies for newborn hearing impairment using a Markov model

**DOI:** 10.3389/fpubh.2025.1498860

**Published:** 2025-05-23

**Authors:** Cheng Wen, Xiaomo Wang, JunTao Shu, Yu Ruan, Jinge Xie, Xiaohua Cheng, Beier Qi, Hui En, Gang Qin, Lihui Huang, Demin Han

**Affiliations:** ^1^Department of Otolaryngology-Head and Neck Surgery, Beijing Tongren Hospital, Capital Medical University, Beijing, China; ^2^Beijing Institute of Otolaryngology, Beijing, China; ^3^Key Laboratory of Otolaryngology Head and Neck Surgery, Ministry of Education, Beijing, China; ^4^Department of Otolaryngology-Head and Neck Surgery, Second Xiangya Hospital, Institute of Otology, Central South University, Changsha, Hunan, China; ^5^Clinical Medical Research Center for Otology in Hunan Province, Changsha, Hunan, China; ^6^Joint Division of Clinical Epidemiology, Affiliated Hospital of Nantong University, School of Public Health of Nantong University, Nantong, China

**Keywords:** newborn hearing screening, deafness, genetic screening, cost-effectiveness, Markov model

## Abstract

**Background:**

Decision-making on how to conduct the concurrent hearing and genetic screening of newborns lacks a health economics basis. To estimate the cost-effectiveness of different newborn hearing screening strategy is necessary.

**Methods:**

A decision tree for a simulated cohort population of 9.56 million newborns was developed: (1) Only universal newborn hearing screening (UNHS); (2) Targeted deafness genetic screening; (3) Concurrent hearing and genetic screening. Markov model was used to evaluate the lifetime horizon (78 years). Cost values were estimated based on medical expenses at Beijing Tongren Hospital and previous studies based on a health system perspective. Health state utility values were represented by Quality-adjusted Life Years (QALYs), costs and utilities were discounted at a rate of 3%. The Incremental Cost-effectiveness Ratio (ICER) was analyzed, along with one-way sensitivity analysis and probability sensitivity analysis.

**Results:**

Compared with only UNHS strategy, the ICER of targeted screening strategy is $ 181.9/QALY, the ICER of concurrent screening is $ 1,563.45/QALY at the discounted rate of 3%. UNHS confirms 21,098 cases of hearing loss, of which 18,666 patients receive early hearing intervention. Concurrent screening confirms 34,244 cases of hearing loss, 26,000 of which receive early hearing intervention. Additionally, 27,036 cases of ototoxicity deafness are avoided. Reducing the cost of genetic screening could enhance the cost-effectiveness of both concurrent screening strategy and targeted genetic screening strategies. At a willingness to pay threshold of 1 time the per capita GDP, at $12,741, the probability of the concurrent screening strategy being cost-effective was 57.6%; at a willingness to pay is three times the per capita GDP, at $38,223, the probability of the concurrent screening strategy being cost-effective was 59.10%.

**Conclusion:**

Both the targeted screening and concurrent screening strategy exhibit good cost-effectiveness compared with only UNHS strategy. The targeted screening strategy is preferable when the willingness to pay is between $181.90/QALY and $1,563.45/QALY, whereas the concurrent screening strategy becomes the preferred choice when the willingness to pay exceeds $1,563.45/QALY.

## Introduction

In 2012, Beijing municipality took the lead in carrying out a genetic screening project for neonatal deafness in China, screening 9 mutation loci of the 4 most common deafness genes in the Chinese population, including c.235delC (p.Leu79Cysfs*3), c.299_300delAT (p.His100Argfs*14), c.176_191del16 (p.Gly59Alafs*18), and c.35delG (p.Gly12Valfs*2) in *GJB2* (MIM: 121011); c.919-2A > G and c.2168A > G (p.His723Arg) in *SLC26A4* (MIM:605646); and m.1555A > G and m.1494C > T of *mtDNA12SrRNA* (MIM: 561000); c.538C > T (p.Arg180*) in *GJB3* (MIM: 603324) (1). In 2013, the Health Development Research Center of the Ministry of Health conducted a health economics evaluation of 200,000 neonates screened for deafness using the nine screening loci of the four genes and found that without conducting deafness genetic screening, saving one labor-year would cost 53,300 yuan, while with deafness genetic screening, such a savings would require a payment of only 5,700 yuan. From the cost–benefit perspective, the cost–benefit ratio of screening is 1:7.27 (2). In addition, through medication guidance and early warning, it is possible to prevent ototoxicity deafness in 560 children. Through targeted education and medication guidance, over 4,000 maternal relatives have been equipped with the necessary knowledge to ensure effective prevention of ototoxicity deafness (2). To date, Beijing has expanded from conducting only universal newborn hearing screening (UNHS) to conducting concurrent hearing and genetic screening of newborns, which has fully demonstrated the important role of deafness genetic screening in determining the molecular etiology of hearing loss, early detection of delayed hearing loss, and ototoxicity deafness. Therefore, early warning systems have been continuously promoted nationwide, and fruitful results have been achieved in the past decade.

Our previous survey on the status of genetic screening for neonatal deafness in several areas of China showed that genetic screening for neonatal deafness has been widely implemented in eastern China but has yet to be gradually promoted in central and western China (3). However, in areas with different levels of economic development, decision-making on how to conduct the concurrent screening of hearing and deafness genes in neonates lacks a health economics basis. Shu *et al.* constructed a decision-tree model to simulate a hypothetical 10-million Chinese newborn cohort with three strategies: no screening, standard newborn hearing screening, combined genetic and hearing screening, found that both standard and combined screening strategies were more effective and more costly than no screening (4). Lv *et al.* assessed the effectiveness and cost-effectiveness of pre-pregnancy deafness screening policies and found that incremental cost-effectiveness ratio (ICER) for reducing deaf newborn births was USD 32,656 per case and USD 1,203,926 per case for increasing one healthy newborn birth (5).

Previous study showed that different deafness gene screening strategies were adopted in different regions of China (3). In some regions, such as Xingtai, Hebei Province, deafness gene screening is carried out for those who fail the newborn hearing screening, that is, targeted deafness genetic screening (6). Although, cost-effectiveness studies on hearing screening and concurrent screening are currently established, there is no cost-effectiveness analysis of strategies for newborn targeted genetic screening. Therefore, to analyze the cost-effectiveness of different hearing loss screening strategies including targeted deafness genetic screening and to provide a health economics basis for the decision-making, the present study is performed from a health system perspective. Based on preliminary survey data and combined with data reported in the literature, this study conducts cost-effectiveness analysis on three screening strategies: only universal newborn hearing screening, concurrent hearing and deafness genetic screening of newborns, and targeted deafness genetic screening.

## Methods

### Data source

Most of the model parameters related to cost, utility, and state transition rate in this study are sourced from our previous studies, and some model parameters are derived from the published literature. The number of neonates is obtained from the Statistical Bulletin of the People’s Republic of China on the National Economic and Social Development in 2022 (7). The model cycle period is chosen according to the 2022 China Statistical Yearbook released by the National Bureau of Statistics in 2022 (8). The mortality rates for different age groups are obtained from the 2020 China Population Census Yearbook published by the National Bureau of Statistics; the data are presented as mortality rates by age and gender in China (from November 1, 2019, to October 31, 2020) (9). The exchange rate between the US dollar and the Chinese yuan for this study is 6.8 in July 2022. The costs involved in this study are mainly direct medical costs, including the expenses for neonate hearing screening, neonate deafness genetic screening, hearing diagnosis, genetic diagnosis, hearing aid fitting, hearing aid adjustment and maintenance, cochlear implantation, and cochlear implant (CI) adjustment.

This study is implemented following the Consolidated Health Economic Evaluation Reporting Standards (CHEERS) 2022: Updated Reporting Guidance for Health Economic Evaluations (10). This study was deemed exempt from ethical review and the need for informed consent by the Beijing Tongren Hospital institutional review board because it is a simulation-based study and not human participant’s research.

### Targeted population

Based on the number of neonates in the Statistical Bulletin of the People’s Republic of China on the National Economic and Social Development in 2022 and the average life expectancy of individuals in the 2022 China Statistical Yearbook, this study simulates the health status of 9.56 million neonates throughout their lifecycles with a simulation period of 78 years and an interval of 1 year. Health status is expressed in QALYs, with a higher value indicating a higher quality of life.

### Model construction

In this study, based on the current status of the investigation of the multicenter concurrent hearing and genetic screening of newborns and the existing literature, a model is constructed using TreeAge Pro 2022 (Supplementary Figure S1). This model is composed of a decision tree and Markov nodes. Three strategies for neonatal hearing and deafness genetic screening are analyzed with the model. Strategy 1 involves only UNHS; i.e., all neonates undergo hearing screening at birth, hereinafter referred to as only hearing screening. Strategy 2 involves the concurrent hearing and genetic screening of newborns; i.e., all neonates receive hearing screening and deafness genetic screening at birth, hereinafter referred to as concurrent screening. Strategy 3 involves all neonates receiving hearing screening at birth, where those who fail hearing screening undergo deafness genetic screening, hereinafter referred to as targeted deafness genetic screening. In the decision tree, each of the three strategies simulates 9.56 million neonates. All three strategies assume that all neonates who fail the hearing screening undergo hearing diagnosis and that all neonates who fail the deafness genetic screening receive hearing and genetic diagnoses.

**Figure 1:**
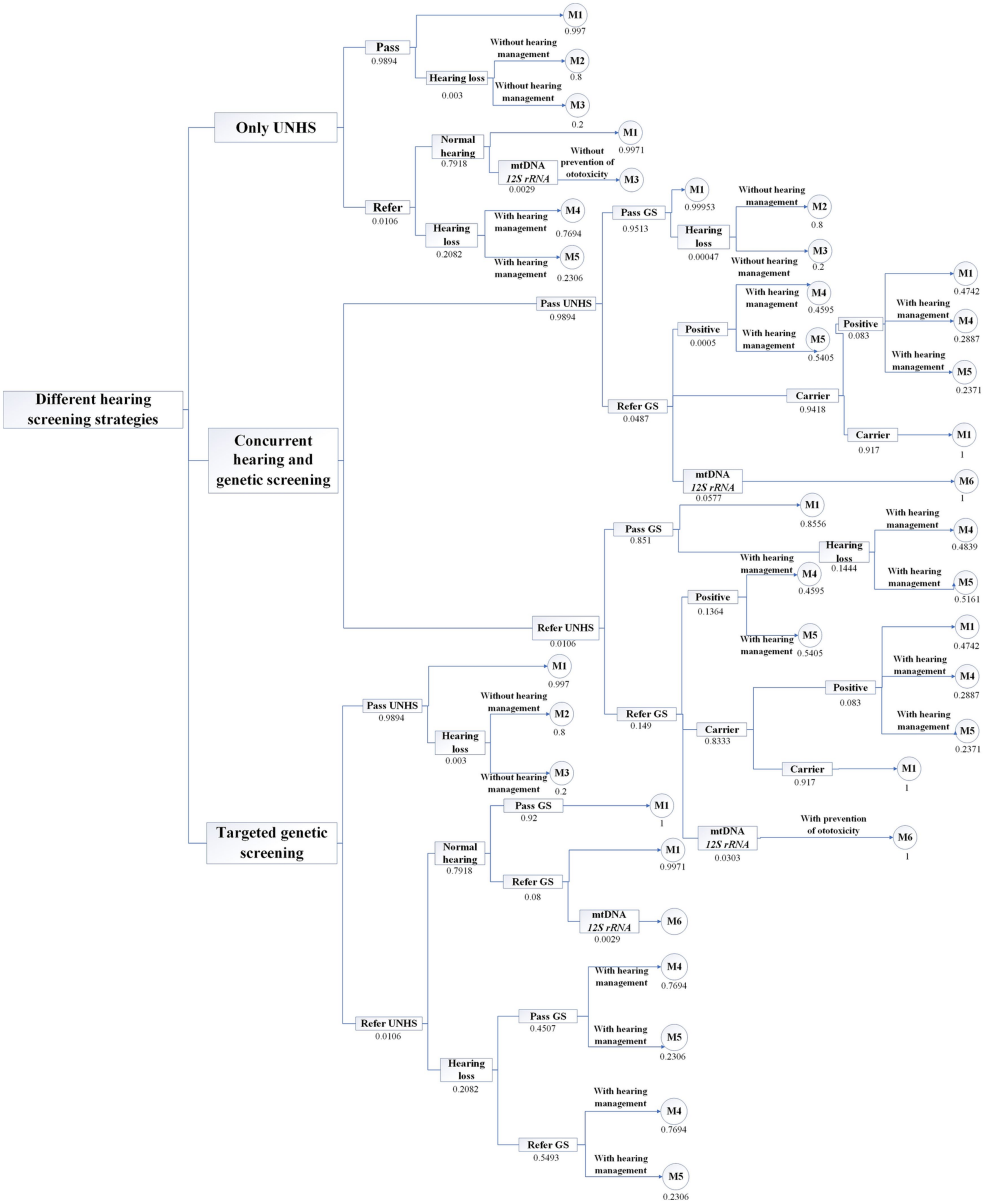
Decision tree structure of 3 hearing screening strategies.

According to the criteria established by the WHO (11), hearing loss severity is classified based on the average hearing threshold of the better ear at 500, 1000, 2000, and 4,000 Hz. Mild to moderate (M/M) hearing loss is defined as 26–60 dB HL, and severe to profound (S/P) hearing loss is defined as 61 dB HL or above (11). The neonates who receive hearing diagnosis consisted of 3 health states: (1) normal hearing, (2) M/M hearing loss, and (3) S/P hearing loss. According to the Markov cycle chain, as shown in Figure 1, the states of those neonates with normal hearing are (1) normal hearing and (2) death. The states of neonates with M/M hearing loss are (1) M/M hearing loss, (2) hearing aid fitting, and (3) death. The states of neonates with S/P hearing loss are (1) S/P hearing loss, (2) cochlear implantation, and (3) death. We assume that 100% of patients diagnosed with M/M hearing loss have received hearing aid fittings and that 50% of those with S/P hearing loss have received CIs (12).

### Model input

The input data for the model are shown in Table 1 (1, 12–29). The parameters for hearing screening and deafness genetic screening in this study are obtained from previous studies. The costs of hearing screening, deafness genetic screening, and deafness genetic diagnosis are estimated based on the medical expenses of Beijing Tongren Hospital. The costs of deafness genetic diagnosis include registration fees, deafness gene sequencing fees, and hearing examination fees. All direct medical costs are calculated in Chinese yuan (RMB) and converted to US dollars at an exchange rate of $1 to RMB 6.8. The costs for the first year of CI and hearing aid fitting, as well as the subsequent annual costs of hearing examination, cochlear or hearing aid adjustment, battery replacement, and device maintenance are estimated based on the previous literature (12). The health utilities are derived from our earlier research and relevant literature. We assume that the health utilities of patients with S/P hearing loss after CI surgery are at least consistent with those of patients with M/M hearing loss.

**Table 1:** Baseline values, range, and reference of model parameters.

Input	Value (range)	Source
*Fail UNHS*	0.0106 (0.0049–0.0124)	Dai P, et al. 2019 (13), Li MT, et al. 2022 (14), and Wen CF, et al. 2023 (15).
Pass genetic screening	0.851 (±20%)	Wen C, et al. 2023 (1).
Fail genetic screening	0.149 (±20%)	Wen C, et al. 2023 (1).
Carrier	0.8333 (±20%)	
Positive	0.1364 (±20%)	
M/M	0.4595 (±20%)	Dai P, et al. 2019 (13).
S/P	0.5405 (±20%)	Dai P, et al. 2019 (13).
mtDNA *12 s rRNA*	0.0303 (±20%)	
*Normal hearing*	0.7918 (±20%)	Dai P, et al. 2019 (13) and Zou L, et al. 2021 (16).
Pass genetic screening	0.92 (±20%)	Yuan Y, et al. 2009 (17).
Fail genetic screening	0.08 (±20%)	Yuan Y, et al. 2009 (17).
mtDNA *12 s rRNA*	0.0029 (±20%)	Wen C, et al. 2023 (1).
Non-mtDNA *12 s rRNA*	0.9971 (±20%)	Wen C, et al. 2023 (1).
*Hearing loss*	0.2082 (±20%)	Dai P, et al. 2019 (13) and Zou L, et al. 2021 (16).
M/M	0.7694 (±20%)	Zou L, et al. 2021 (16).
S/P	0.2306 (±20%)	Zou L, et al. 2021 (16).
Pass genetic screening	0.4507 (±20%)	Yuan Y, et al. 2009 (17).
Fail genetic screening	0.5493 (±20%)	Yuan Y, et al. 2009 (17).
*Pass hearing screening*	0.9894 (±20%)	Dai P, et al. 2019 (13).
Pass genetic screening	0.9513 (±20%)	Wen C, et al. 2023 (1).
Fail genetic screening	0.0487 (±20%)	Wen C, et al. 2023 (1).
Carrier	0.9418 (±20%)	Wen C, et al. 2023 (1).
Positive	0.0005 (±20%)	Wen C, et al. 2023 (1).
M/M	0.4595 (±20%)	
S/P	0.5405 (±20%)	
mtDNA *12 s rRNA*	0.0577 (±20%)	Wen C, et al. 2023 (1).
Normal hearing	0.997 (±20%)	Yousefi J, et al. 2013 (18).
*Hearing loss*	0.003 (±20%)	Yousefi J, et al. 2013 (18).
M/M	0.8 (±20%)	Dai P, et al. 2019 (13).
S/P	0.2 (±20%)	Dai P, et al. 2019 (13).
*Pass UNHS and genetic screening*		
Normal hearing	0.99953 (±20%)	Li L, et al. 2012 (19) and Lü J, et al. 2011 (20).
Hearing loss	0.00047 (±20%)	Li L, et al. 2012 (19) and Lü J, et al. 2011 (20).
*Fail UNHS and pass genetic screening*		
Normal hearing	0.8556 (±20%)	Li MT, et al. 2022 (14) and Zhu QW, et al. 2021 (21).
Hearing loss	0.1444 (±20%)	Li MT, et al. 2022 (14) and Zhu QW, et al. 2021 (21).
M/M	0.4839 (±20%)	Li MT, et al. 2022 (14) and Zhu QW, et al. 2021 (21).
S/P	0.5161 (±20%)	Li MT, et al. 2022 (14) and Zhu QW, et al. 2021 (21).
Carrier after sequencing	0.917 (±20%)	Cui QJ, et al. 2015 (22) and Zhao XL, et al. 2019 (23).
*Positive after sequencing*	0.083 (±20%)	Cui QJ, et al. 2015 (22) and Zhao XL, et al. 2019 (23).
NH	0.4742 (±20%)	Cui QJ, et al. 2015 (22) and Zhao XL, et al. 2019 (23).
M/M	0.2887 (±20%)	Cui QJ, et al. 2015 (22) and Zhao XL, et al. 2019 (23).
S/P	0.2371 (±20%)	Cui QJ, et al. 2015 (22) and Zhao XL, et al. 2019 (23).
*Cost estimates, USD*		
Genetic screening	34.1 (18.9–144)	Estimated
Hearing screening	11.93 (±20%)	Huang L, et al. 2012 (24).
Hearing diagnosis	170 (90–310)	Huang L, et al. 2012 (24).
Genetic diagnosis	335 (±20%)	Estimated
Hearing aids (First year)	1960 (1740–2,180)	Huang L, et al. 2012 (24).
Hearing aids (annual)	302 (220–350)	Qiu J, et al. 2017 (25).
Cochlear implant (First year)	30,000 (22500–45,000)	Qiu J, et al. 2017 (25).
Cochlear implant (annual)	1,500 (750–2,250)	Qiu J, et al. 2017 (25).
*Health state QoL weights*		
Normal hearing	1	
M/M	0.8 (0.78–0.82)	Crowson MG, et al. 2017 (26).
S/P	0.54 (0.52–0.56)	Crowson MG, et al. 2017 (26).
Hearing aids	0.96 (0.768–1)	Montes F, et al. 2017 (27).
Cochlear implant	0.8 (0.78–0.82)	Crowson MG, et al. 2017 (26).
Death rate	Rates vary by age group from 0 to 78 years	http://www.stats.gov.cn/sj/pcsj/rkpc/7rp/indexch.htm
Cochlear implant ratio of S/P	0.5 (0.2–1.0)	Chen K, et al. 2020 (12) and Gantt S, et al. 2020 (28).
*Discounted rate*	3, 5%	2020 Guidelines for Pharmacoeconomic Evaluation in China (29).

### Cost-effectiveness analysis

In this study, based on a decision tree model, the cost-effectiveness of different strategies is analyzed from a sociological perspective. Cost-effectiveness analysis employs primarily TreeAge Pro to calculate and compare the cost-effectiveness of different screening strategies, that is, the cost per QALY. In addition, the ICER explains the increased cost for every additional 1 QALY, with reference to the screening strategy with the lowest cost-effectiveness value. According to the Guide to Cost-effectiveness Analysis released by the WHO in 2003, a cost-effectiveness scenario is considered when the ICER falls within 1 to 3 times the per capita national gross domestic product (GDP) (30). According to the Statistical Bulletin of the People’s Republic of China on National Economic and Social Development in 2022, China’s annual per capita GDP in 2022 was 85,698 RMB, approximately equivalent to $12,458 (7).

### One-way sensitivity analysis

This study performs a one-way sensitivity analysis to assess the impact of changes in hearing screening probability indicators, deafness genetic screening probability pathogenicity, cost parameters, and transition probability on cost-effectiveness. Referring to previous studies, a 95% confidence interval is set for health utilities (12, 26), the variation range of transition rates is set to 20% (12), and the variation ranges of other parameters depend on the degree of uncertainty in terms of the basic values.

At present, Beijing, Shanghai, Guangdong, Shandong, Jilin, Liaoning, Hunan, Henan, Zhejiang, Heilongjiang and other provinces and cities have issued relevant documents on cochlear implantation and auditory and speech rehabilitation assistance policies, such as the Implementation Rules for the Management of the Cochlear Implant Rehabilitation Assistance Project for Children with Hearing Impairment in Shandong Province, announcement on the launching of the cochlear implant donation project of “Hearing reconstruction and hearing enlightenment action” in 2023, and so on. In the future, the cochlear implantation rate of children with severe to profound hearing loss may be improved. Therefore, this study analyzes the cost-effectiveness of CIs of between 20 and 100% through one-way sensitivity analysis.

### Probability sensitivity analysis

Probability sensitivity analysis is used mainly in the context of uncertainty analysis to explore the impact of simultaneous changes in multiple parameters on the results. In this study, the probability and utility values follow a beta distribution, the cost values follow a gamma distribution, and the mean is the baseline value. Through random sampling from the probability distribution, all the parameters of the transformed distribution are input into the software for 1,000 Monte Carlo simulations to observe the influence of the uncertainty factors on the model as well as the acceptability curve. The willingness-to-pay curve can be used to observe the probabilities of different screening strategies for neonate hearing screening being cost-effective at different willingness-to-pay levels, considering multiple uncertain factors.

### Statistical analysis methods

The decision tree model is created by using TreeAge Pro 2022 (TreeAge, Inc., United Statets) to perform cost-effectiveness calculations, cost-effectiveness analysis, one-way sensitivity analysis, and probability sensitivity analysis.

## Results

### Basic analysis results

The only UNHS strategy confirms 21,098 cases of hearing loss, of which 18,666 patients receive early hearing intervention. The concurrent hearing and genetic screening of newborns confirms 34,244 cases of hearing loss, 26,000 of which receive early hearing intervention. Additionally, a total of 27,036 cases of ototoxicity deafness are avoided. Hearing screening is performed for all neonates. All 101,336 neonates who fail to pass the screening undergo deafness genetic screening, which identifies 21,098 cases of hearing loss, of whom 18,666 receive early hearing intervention. In addition, ototoxicity deafness is avoided in 19 cases. The screening results are presented in Table 2.

**Table 2:** Relative performance of 3 screening strategies (*n*, 95% CI).

Characteristic	Only UNHS		Concurrent screening		Targeted genetic screening	
	*N* (*n*, 95% CI)	%	*N* (*n*, 95% CI)	%	*N* (*n*, 95% CI)	%
Newborns genetic screened	0	0.00	9,560,000	100.00	101,336	1.06
Hearing loss diagnosed	21,098 (20,815 ~ 21,385)	0.22	34,224 (33,863 ~ 34,588)	0.36	21,098 (20,815 ~ 21,385)	0.22
Hearing aids	16,233 (15,984 ~ 16,484)	0.17	17,775 (17,515 ~ 18,038)	0.19	16,233 (15,984 ~ 16,484)	0.17
Cochlear implant	2,433 (2,337 ~ 2,531)	0.03	8,225 (8,049 ~ 8,404)	0.09	2,433 (2,337 ~ 2,531)	0.03
Ototoxicity deafness avoided	/	/	27,036 (26,715 ~ 27,395)	0.28	19 (11 ~ 30)	0.0002

As shown in Table 3, in the absence of discounting, the ICER of targeted genetic screening strategy is $697.32/QALY, ICER of concurrent screening strategy is $2,040/QALY. When both cost and utility are discounted at a rate of 3%, the ICER of targeted genetic screening strategy is $ 181.9/QALY, ICER of concurrent screening strategy is $1,563.45/QALY. When both cost and utility are discounted at a rate of 5%, the ICER of targeted genetic screening strategy is $62.62/QALY, ICER of concurrent screening strategy is $1659.31/QALY. The ICERs are all lower than the level of 1–3 times the per capita GDP recommended by the WHO.

**Table 3:** Cost-effectiveness analysis of 3 screening strategies applied to 9.56 million newborns.

Screening strategy	Cost (USD)	Effectiveness, QALY	Incremental cost, USD	Incremental effectiveness, QALY	ICER (USD/QALY)
Cost and effectiveness undiscounted (0%)					
Only UNHS	59,887,757,346	58,338,083,489	/	/	
Targeted genetic screening	60,341,164,828	58,338,733,708	453,407,482	650,218	697.32
Concurrent hearing and genetic screening	163,201,457,335	58,388,718,773	103,313,699,989	50,635,283	2040
Targeted genetic screening	60,341,164,828	58,338,733,708	/	/	
Concurrent hearing and genetic screening	163,201,457,335	58,388,718,773	102,860,292,507	49,985,065	2057.82
Cost and effectiveness discounted 3%					
Only UNHS	7,716,562,300	9,017,588,299	/	/	
Targeted genetic screening	7,734,866,106	9,017,688,924	18,303,806	100,625	181.9
Concurrent hearing and genetic screening	18,373,714,683	9,024,404,733	10,657,152,383	6,715,809	1563.45
Targeted genetic screening	7,734,866,106	9,017,688,924	/	/	
Concurrent hearing and genetic screening	18,373,714,683	9,024,404,733	10,638,848,577	6,816,434	1,584
Cost and effectiveness discounted 5%					
Only UNHS	3,123,735,099	3,935,941,946	/	/	
Targeted genetic screening	3,126,489,612	3,935,985,931	2,754,513	43,985	62.62
Concurrent hearing and genetic screening	7,136,531,952	3,938,360,294	4,012,796,853	2,418,347	1659.31
Targeted genetic screening	3,126,489,612	3,935,985,931	/	/	
Concurrent hearing and genetic screening	7,136,531,952	3,938,360,294	4,010,042,340	2,374,362	1688.89

### One-way sensitivity analysis

**Figure 2:**
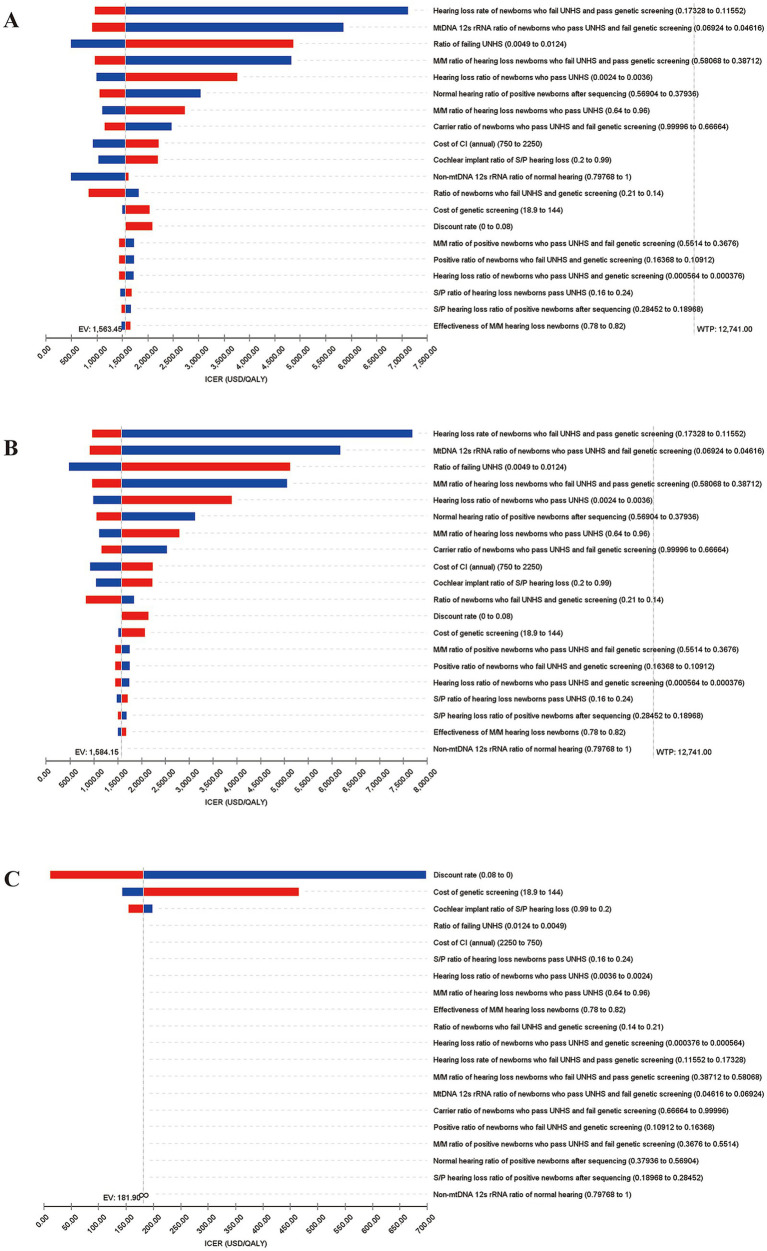
Tornado diagrams for 1-Way sensitivity analysis of incremental cost-effectiveness ratios (ICERs). The top 20 variables and the variation range of the ICER are shown. **(A)** Analysis for concurrent screening vs. only UNHS; **(B)** Analysis for concurrent screening vs. targeted genetic screening; **(C)** Analysis for targeted genetic screening vs. only UNHS.

One-way sensitivity analysis is conducted on the 56 variables included in the model. The top 20 variables and the variation range of the ICER are shown in Table 4. Tornado diagrams are presented in Figure 2. Reduce ratio of failing UNHS, hearing loss ratio of newborns who pass UNHS, M/M ratio of hearing loss newborns who pass UNHS, cost of CI (annual), cost of genetic screening, cochlear implant ratio of S/P hearing loss, could improve the cost utility of concurrent screening strategy. Meanwhile, increase hearing loss ratio of newborns who fail UNHS and pass genetic screening, MtDNA 12srRNA ratio of newborns who pass UNHS and fail genetic screening, ratio of newborns who fail UNHS and genetic screening, M/M ratio of positive newborns who pass UNHS and fail genetic screening, positive ratio of newborns who fail UNHS and genetic screening, hearing loss ratio of newborns who pass UNHS and genetic screening, S/P hearing loss ratio of positive newborns after sequencing, could improve the cost utility of concurrent screening strategy. Reduce the cost of genetic screening and increase cochlear implant ratio of S/P hearing loss could improve the cost utility of targeted genetic screening strategy.

**Table 4:** 1-Way sensitivity analysis of concurrent hearing and genetic screening.

Characteristic	Range	ICER range (USD/QALY)		Targeted genetic screening vs. only UNHS
		Concurrent screening vs. only UNHS	Concurrent screening vs. targeted genetic screening	
Hearing loss ratio of newborns who fail UNHS and pass genetic screening	0.11552 ~ 0.17328	959.95 ~ 7111.39	7675.13 ~ 966.37	181.90
MtDNA *12 s rRNA* ratio of newborns who pass UNHS and fail genetic screening	0.04616 ~ 0.06924	902.79 ~ 5840.15	908.98 ~ 6170.48	181.90
Ratio of failing UNHS	0.0049 ~ 0.0124	482.52 ~ 4857.97	483.18 ~ 5120.33	181.90
M/M ratio of hearing loss newborns who fail UNHS and pass genetic screening	0.38712 ~ 0.58068	954.64 ~ 4280.97	961.47 ~ 5049.12	181.90
Hearing loss ratio of newborns who pass UNHS	0.0024 ~ 0.0036	986.51 ~ 3766.06	994.07 ~ 3898.21	181.90
Normal hearing ratio of positive newborns after sequencing	0.37936 ~ 0.56904	1052.79 ~ 3039.42	1061.53 ~ 3123.89	181.90
M/M ratio of hearing loss newborns who pass UNHS	0.64 ~ 0.96	1096.52 ~ 2722.00	1106.09 ~ 2790.06	181.90
Carrier ratio of newborns who pass UNHS and fail genetic screening	0.66664 ~ 0.99996	1146.28 ~ 2471.87	1156.78 ~ 2526.87	181.90
Cost of CI (annual)	750 ~ 2,250	919.44 ~ 2207.46	930.49 ~ 2237.81	181.90
Cochlear implant ratio of S/P hearing loss	0.2 ~ 0.99	1026.59 ~ 2201.47	1040.79 ~ 2226.88	155.04 ~ 198.35
Non-mtDNA 12 s rRNA ratio of normal hearing	0.79768 ~ 1	487.96 ~ 1615.27	1584.15 ~ 1615.27	−153.83 ~ 28.89
Ratio of newborns who fail UNHS and genetic screening	0.1192 ~ 0.1788	828.12 ~ 1822.49	832.81 ~ 1851.59	181.90
Cost of genetic screening	18.9 ~ 144	1497.01 ~ 2043.81	1517.31 ~ 2067.46	142.67 ~ 465.56
Discount rate	0 ~ 0.08	2040.35 ~ 2088.72	2057.82 ~ 2143.49	11 ~ 697.32
M/M ratio of positive newborns who pass UNHS and fail genetic screening	0.3676 ~ 0.5514	1429.44 ~ 1733.84	1446.11 ~ 1760.30	181.90
Positive ratio of newborns who fail UNHS and genetic screening	0.10912 ~ 0.16368	1436.81 ~ 1727.29	1453.45 ~ 1753.91	181.90
Hearing loss ratio of newborns who pass UNHS and genetic screening	0.000376 ~ 0.000564	1438.11 ~ 1712.72	1455.40 ~ 1737.89	181.90
S/P ratio of hearing loss newborns pass UNHS	0.16 ~ 0.24	1458.63 ~ 1684.50	1476.46 ~ 1708.78	181.90
S/P hearing loss ratio of positive newborns after sequencing	0.18968 ~ 0.28452	1489.67 ~ 1665.90	1506.48 ~ 1692.54	181.90
Effectiveness of M/M hearing loss newborns	0.78 ~ 0.82	1651.35 ~ 1484.43	1502.95 ~ 1674.63	181.90

### Probability sensitivity analysis

**Figure 3:**
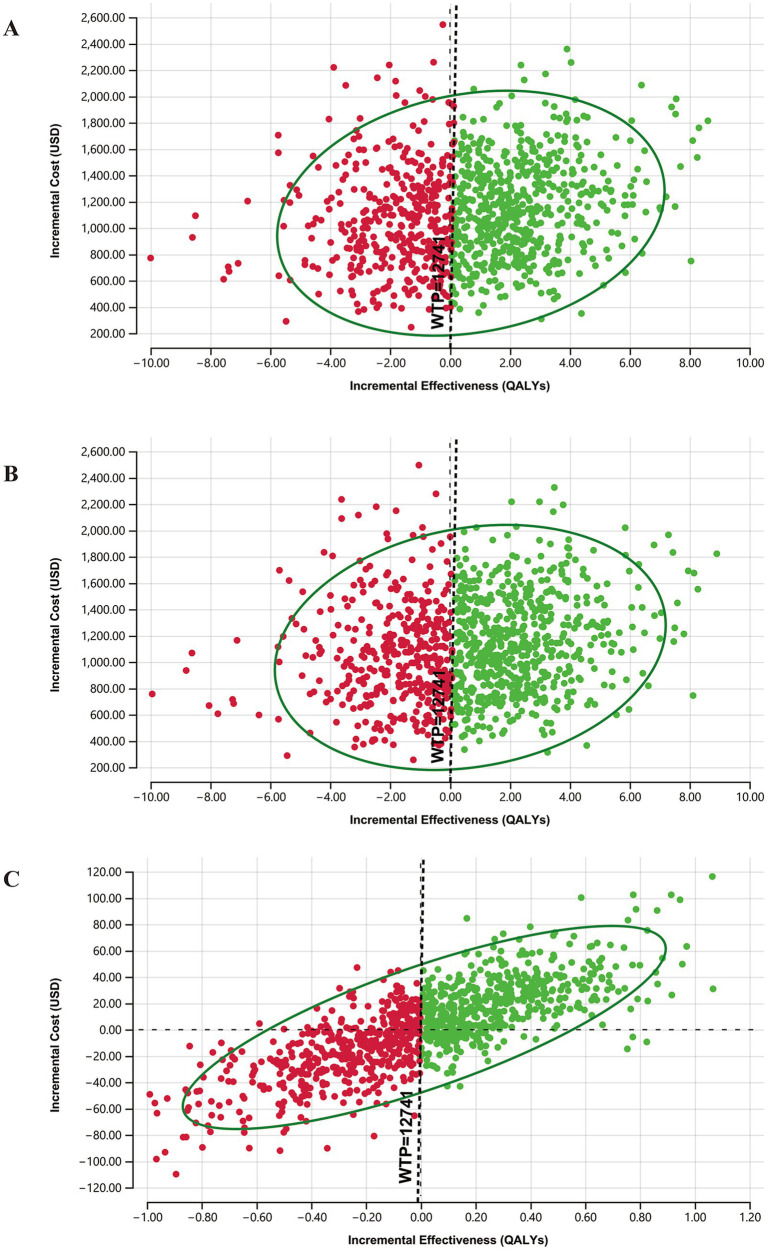
Results of probability sensitivity analysis. **(A)** ICE scatterplot diagrams for concurrent screening vs. only UNHS; **(B)** Analysis for concurrent screening vs. targeted genetic screening; **(C)** Analysis for targeted genetic screening vs. only UNHS.

As shown in Figures 3A,B, 60.1 and 59.9% of the scatters (green) are distributed in the first quadrant, respectively, indicating that the concurrent screening strategy is more costly and has better health output than only UNHS and targeted genetic screening. As can be seen in Figure 3C, 41.7% of scatters are distributed in the first quadrant, and 37.2% are distributed in the third quadrant, indicating that the targeted genetic screening strategy results in better health outcomes but also involves higher costs and that reducing costs may worsen health outcomes in this context.

**Figure 4:**
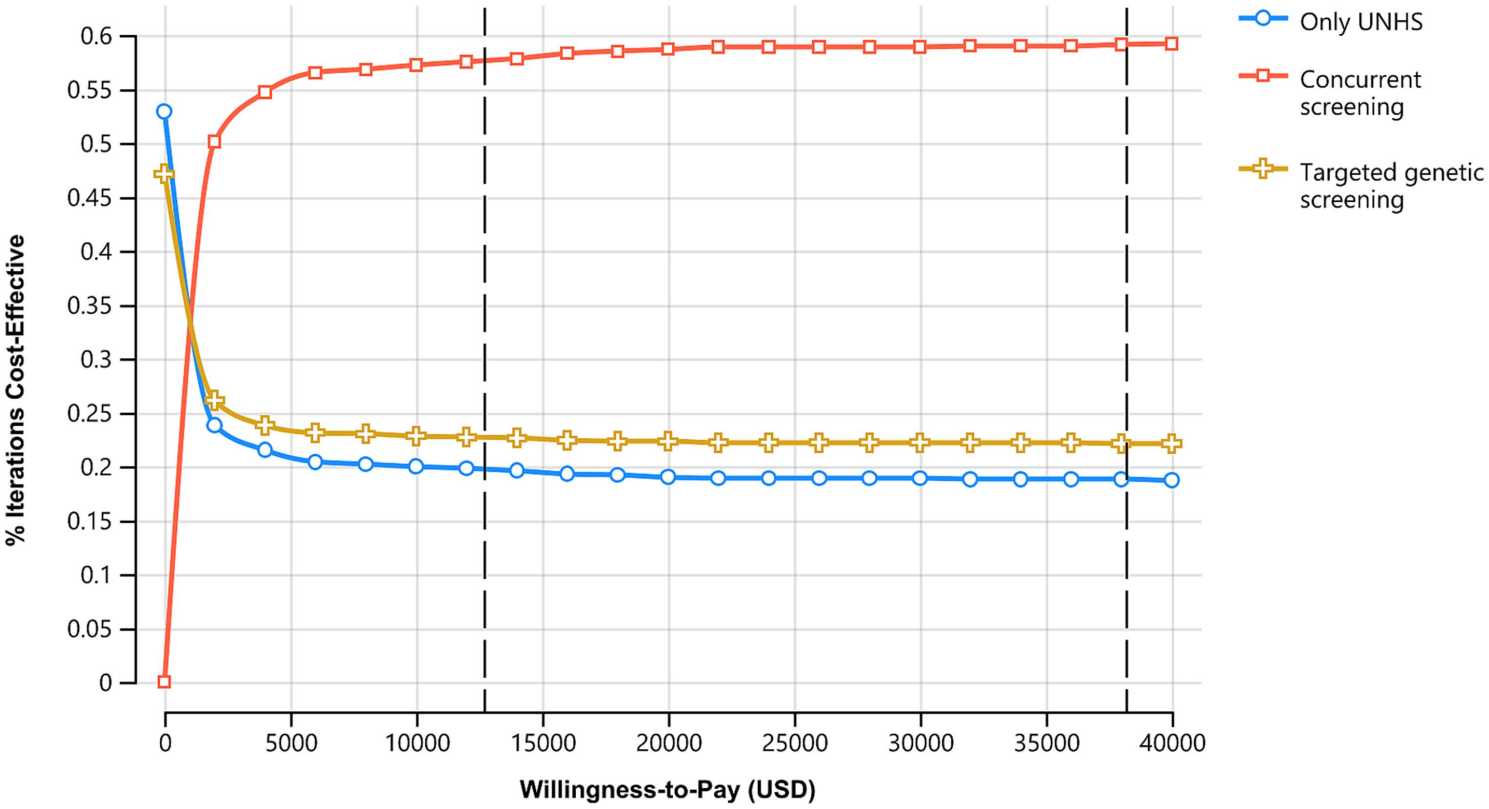
Acceptability curve of 3 hearing loss screening strategies. With the increase in the WTP, probabilities being cost-effective of only UNHS and targeted genetic screening decrease while concurrent screening increases.

The cost-effectiveness acceptability curves are shown in Figure 4. With the increase in the willingness-to-pay values, the probabilities of only UNHS strategy and targeted genetic screening strategy being cost-effective gradually decrease, while that of concurrent screening strategy being cost-effective gradually increases. The targeted genetic screening strategy is preferable when the willingness to pay is between $181.90 per QALY and $1,563.45 per QALY, whereas the concurrent screening strategy becomes the preferred choice when the willingness to pay exceeds $1,563.45 per QALY. When the willingness to pay is 1 time the per capita GDP of US $12,741, the probability of the only UNHS strategy being cost-effective is 19.7%, that of the concurrent screening strategy being cost-effective is 57.6%, and that of the targeted genetic screening strategy being cost-effective is 22.7%. When the willingness to pay is 3 times the per capita GDP of US $ 38,223, the probability of the only UNHS strategy being cost-effective is 18.8%, that of the concurrent screening strategy being cost-effective is 59.1%, and that of the targeted genetic screening strategy being cost-effective is 22.1%.

## Discussion

This study finds that compared to the only UNHS strategy, the targeted genetic screening strategy and the concurrent screening strategy have better degrees of cost-effectiveness. The screening strategy can be chosen based on willingness to pay, which, when greater than $1,563.45 per QALY, the strategy of concurrent screening strategy should be chosen; when the willingness to pay is between $181.90 per QALY and $1,563.45 per QALY, the targeted genetic screening strategy should be considered. The following discussion covers four aspects: the epidemiology of hearing loss in neonates and children, the advantages of hearing loss and deafness genetic screening in neonates, the factors affecting the ICER, and the contributions and limitations of this study.

### Epidemiology of hearing loss in neonates and children

According to the World Report on Hearing released by the WHO in 2021, approximately 1.5 billion people worldwide, including 34 million children, suffer from various degrees of hearing loss. The global prevalence of moderate or above hearing loss is increasing with age and includes approximately 2‰ of neonates, 4‰ of infants under 1 year, 1% of infants 1–4 years old, 1.5% of children 5–9 years old, and 1.7% of children 10–14 years old (31).

The prevalence of permanent bilateral sensorineural hearing impairment in neonates is approximately 1.331‰ in those developed countries that have implemented universal newborn hearing screening programs, such as the US, and approximately 19‰ in those countries without universal newborn hearing screening programs, such as those countries in sub-Saharan Africa (32). Regarding the occurrence of hearing impairment in children in other countries, according to the 2020 report of the US Centers for Disease Control and Prevention, the prevalence of disabling hearing loss in children and adolescents in Europe and North America is approximately 1‰, and the prevalence of disabling hearing loss in 8-year-old children in the US is approximately 1.4‰ (33).

Regarding the incidence of neonatal hearing impairment in China, a total of 180,469 neonates were screened in Beijing in 2019, and the prevalence of hearing loss was 1.31‰ (13). In 2023, the results of newborn hearing screening in Hainan Province showed that the prevalence of hearing impairment among 94,118 normal neonates was 4.1‰ and that among 2,536 high-risk neonates was 1.66% (15). Regarding the incidence of hearing impairment in children in China, the first national sample survey on disabled people in 1987 showed that the prevalence of hearing impairment in children aged 0–7 years was 1.99‰ (34). The second sample survey of persons with disabilities in 2006 revealed that among children under 17 years old, 221.5 thousand had hearing impairment alone and 359.3 thousand had multiple disabilities including hearing impairment (35). In 2016, Hu et al. (36) reported that the prevalence of hearing loss in the 0-to-14-year-old group in four provinces in China was 0.85%.

It has been reported that genetic factors cause more than 50% of hearing loss in neonates and approximately 40% of hearing loss in children (31). Therefore, newborn hearing screening and deafness genetic screening can help achieve early detection and intervention in terms of hearing loss and maximumly help children with hearing loss integrate into mainstream society.

### Advantages of hearing and deafness genetic screening in neonates

In 2012, Beijing adopted the hereditary deafness gene detection chip independently developed in China and took the lead in launching a large-scale neonatal deafness genetic screening project. This project involved deafness genes screening using neonatal heel blood, followed by genetic diagnosis and counseling for those neonates with positive screening results. Thus, the concurrent screening strategy in China has gradually expanded nationwide and progressed toward a mature and developed stage. This situation has significant clinical guidance implications for the early detection and intervention of common deafness mutations in neonates and the early warning regarding carriers of ototoxicity deafness genes.

The WHO has made a conservative estimate of the return on investment owing to newborn hearing screening in low-, middle-, and high-income settings. In low- and middle-income settings, approximately 1.67 international dollars are gained for every international dollar invested in neonate hearing screening. In high-income settings, a return of approximately 6.53 international dollars is obtained for every international dollar invested (31). Huang et al. (24) reported that universal newborn hearing screening in economically developed areas and hearing screening of the targeted population in underdeveloped areas demonstrate favorable levels of cost-effectiveness in 2012. In 2020, Chen et al. (12) used a Markov model to analyze the cost-effectiveness of different screening strategies for congenital cytomegalovirus infection (cCMVi) and recommended the implementation of universal cCMVi screening for neonates. Thus far, research comparing the levels of cost-effectiveness of different strategies for neonatal hearing and deafness genetic screening has rarely been reported.

This study analyzes the cost-effectiveness of the targeted genetic screening strategy and concurrent screening strategy compared to the newborn hearing screening strategy alone, which can provide a basis for the selection of screening strategies in different economically developed regions. In this study, the ICER for the targeted genetic screening strategy is $181.9/QALY, and that for the combined screening strategy for neonatal hearing and deafness genes is $1,563.45/QALY, both of which are, thus, shown to be cost-effective. According to previous research, the lifetime indirect costs associated with hearing loss may be 2 to 5 times the direct costs (37). Hearing loss that is not intervened in can lead to an annual global economic loss of $980 billion, including healthcare expenses, educational support, productivity loss, and social costs (38). The WHO has called for an additional investment of $1.33 per person per year in the health system to improve the diagnosis, treatment, and rehabilitation of ears and hearing loss; this investment may benefit nearly 1.5 billion people worldwide, avoid the loss of 130 million disability-adjusted life years, and bring about gains of more than $2.4 trillion (31). The results of this study show that compared with the strategy of newborn hearing screening alone, the effectiveness of the screening strategy for deafness genes can increase by 100,625 QALYs, and concurrent screening strategy can increase by 6,715,809 QALYs, both of which can benefit neonates with hearing loss. In addition, deafness genetic screening in the neonatal period helps clarify the cause of hearing loss and enables super early intervention in neonates with hereditary, permanent hearing loss. Considering the expected lifetime benefits, this approach may be more cost-effective than other approaches.

### Factors affecting the ICER

The results of one-way sensitivity analysis show that increases in the ratio of failing UNHS, hearing loss ratio of newborns who pass UNHS, cost of CI (annual), and cost of genetic screening raise the ICER of the concurrent screening strategy. Moreover, decreases in the discount rate and cochlear implant ratio of S/P hearing loss and an increase in the cost of genetic screening raise the ICER of the targeted genetic screening strategy. Therefore, implementing quality control measures in the UNHS project to reduce the false-positive and screening omission rates can effectively improve the cost-effectiveness of the concurrent screening strategy. Reducing maintenance costs after cochlear implantation, such as battery and sound processor replacement costs, can also improve the cost-effectiveness of the concurrent screening strategy. Furthermore, lowering the cost of deafness genetic screening improves the cost-effectiveness of the concurrent screening strategy and the targeted genetic screening strategy. Beijing has been conducting government-subsidized genetic screening for hearing loss since April 2012, providing free screening for all newborns. As of now, over 2 million newborns have received free gene screening, and other regions can draw on this successful experience.

The cochlear implantation ratio in patients with S/P hearing loss is closely related to the ICER of the targeted genetic screening strategy. In high-income countries, almost all children with S/P hearing loss undergo cochlear implantation on at least one ear (12). The WHO conducted a conservative assessment of the cost-effectiveness of unilateral cochlear implantation. The estimation based on the actual cost for the high-income population showed a return of 2.59 international dollars for every US dollar invested, resulting in a value of $38,153 saved per DALY per person. Among the lower-middle-income group, the return on investment is 1.46 international dollars, leading to a total saving of $6,907 per DALY per person. For the middle- and high-income populations, the return on investment is estimated to be 4.09 international dollars, resulting in a savings of $24,161 per DALY per person (31).

In China, the rate of cochlear implantation in children with S/P hearing loss may be related to the assistance policy in place. In August 2023, the General Office of the National Health Commission issued the Notice on Printing and Distributing the Plan for Improving the Prevention and Control Capacity of Birth Defects (2023–2027) (NHC General Office Women and Children Document [2023] No. 9), which clearly states that the intervention rate for neonatal hearing impairment should reach 90% within 6 months. At present, provinces and cities such as Beijing, Shanghai, Guangdong, Shandong, Jilin, Liaoning, Hunan, Henan, Zhejiang, and Heilongjiang have all issued relevant guidelines on assistance policies for cochlear implantation and auditory-speech rehabilitation, with the specific scope of such assistance varying across regions. To further advance the reform of centralized volume-based procurement for high-value medical consumables, on November 29, 2024, the Chinese National Healthcare Security Administration released “the Notice on the National Centralized Procurement of Cochlear Implant and Peripheral Vascular Stent Categories of Medical Consumables (Guo Hao Lian Cai [2024] No. 2).” Starting in March 2025, several provinces and regions, including Beijing, Shanghai, Hunan, and Shaanxi, have implemented the latest cochlear implant centralized procurement policies, reducing the price per cochlear implant from the original range of 200,000 to 300,000 RMB to a uniform price of 50,000 RMB. This may further increase the cochlear implantation rate, thereby improving the cost-effectiveness of targeted genetic screening.

### Contribution and limitations of this study

The contribution of this study is that a decision Markov model is established based on data from the study of the current situation of neonatal hearing and deafness genetic screening in Beijing. The model simulates the health utility values throughout the entire lifecycle of neonates, and the main model parameters are derived from studies conducted on the Chinese population. This approach makes the evaluation of screening strategies more relevant, comprehensive, and objective. The research results provide an effective reference for the development of early screening strategies for neonatal hearing impairment in different economically developed regions in China. This study performs one-way sensitivity analysis on all parameters to clarify the impact of the changes in each parameter on the degree of cost-effectiveness, which provides a scientific basis on which to improve the cost-effectiveness of early screening strategies for neonatal hearing impairment.

The limitation of this study is that the model does not reflect the QALYs that early etiological diagnosis can be achieved by deafness gene screening in the neonatal period, and the impact on speech development may be improved. Due to the lack of parameter sources, this study did not consider the impact of neonatal guardians’ gender, age, education and other factors on the cognition of different screening strategies. Only direct medical costs were included in the cost, and cost-effectiveness analysis was conducted from the perspective of health care. In addition, the lack of granular regional economic data including urban vs. rural cost variations and potential biases from excluding false-positive or false-negative screening outcomes including unnecessary diagnostics or missed interventions were another limitation of the study. This results in different economically developed regions needing to consider both local screening costs and current screening situations when selecting screening strategies for comprehensive consideration. In the future, we should incorporate indirect costs into our analysis. Furthermore, it is essential to conduct a comparative analysis of the varying screening costs across different regions and include the evaluation of false positives and false negatives in the cost-effectiveness analysis. This comprehensive approach will provide a more robust foundation for decision-making in diverse regional contexts.

## Data Availability

The original contributions presented in the study are included in the article/Supplementary material, further inquiries can be directed to the corresponding authors.
