# Supplementary material for: Estimated cost-effectiveness of early screening strategies for newborn hearing impairment using a Markov model
Source: Front Public Health. 2025 May 23;13:1498860. doi: 10.3389/fpubh.2025.1498860 (PMC12141332; doi:10.3389/fpubh.2025.1498860)
Supplement: Supplementary file 1 [file Supplementary_file_1.docx]

**Supplementary Data**

**Fig. S1** State-transition diagram
